# The effect of risk communication on preventive and protective Behaviours during the COVID-19 outbreak: mediating role of risk perception

**DOI:** 10.1186/s12889-020-10125-5

**Published:** 2021-01-06

**Authors:** Seyed Taghi Heydari, Leila Zarei, Ahmad Kalateh Sadati, Najmeh Moradi, Maryam Akbari, Gholamhossin Mehralian, Kamran Bagheri Lankarani

**Affiliations:** 1 Health Policy Research Center, Institute of Health, Shiraz University of Medical Sciences, Shiraz, Iran; 2 Department of Social Sciences, Yazd University, Yazd, Iran; 3 Health Management and Economics Research Center, Iran University of Medical Sciences, Tehran, Iran; 4 Department of Pharmacoeconomics and Pharma management, Shahid Beheshti University of Medical Sciences, Tehran, Iran

**Keywords:** SARS-CoV2, Risk perception, Risk communication, Behaviors, Protective measures, Preventive measures

## Abstract

**Background:**

The COVID-19 outbreak is a global pandemic, during which the community preventive and protective behaviors play a crucial role in the containment and control of infection. This study was designed to contribute to the existing knowledge on how risk communication (RC) and risk perception (RP) affect protective and preventive behaviors (PPB) during the COVID-19 outbreak.

**Methods:**

The required data were extracted from a national online survey of Iranian adults aged 15 and older during March 15–19, 2020 (*n*=3213). Data analysis was performed using structural equation modeling.

**Results:**

The study findings reveal that RC has direct and indirect positive effects on PB. Furthermore, this study also provides new evidence indicating that RP mediates the relationship between RC and PB and there is a two-way relationship between RC and RP. These interactions may have impact on risk communication strategies which should be adopted during this pandemic.

**Conclusion:**

The study findings have remarkable implications for informing future communications as well as interventions during this ongoing outbreak and subsequent national risk events.

**Supplementary Information:**

The online version contains supplementary material available at 10.1186/s12889-020-10125-5.

## Background

The new coronavirus (COVID-19) is officially a pandemic [1]. In spite of strict universal control and quarantine attempts, the outspread of COVID-19 is continuously rising [2]. This pandemic is affecting all sectors of societies, even for those who were not affected by the virus directly [3]. Given the multilateral effects of COVID-19 and its alarming consequences, social concern has become a very complicated issue. Accordingly, an effective risk communication is essential not only to limit its morbidity and mortality but also to minimize the damages posed on the national economies and public health infrastructure [4]. This fact is prominent in Iran as a country with the ever-highest politically-induced sanctions [5].

According to the CDC’s manual of crisis and emergency risk communication (CERC), non-pharmaceutical measures, like quarantine, would be an extreme public health scenario once an episode of a severe communicable disease occurs [6]. Iran as the hardest-hit country in the Middle East, is struggling its third wave of the COVID-19 epidemic [7, 8]. The virus began to spread on February 19 and it had gotten a high mortality rate and lead to 1,158,384 confirmed cases and 53,625 deaths in December 2020 [9]. At the commencement of the outbreak, the country immediately delivered some measures to prevent spread of the COVID-19 such as closing all educational as well as recreational centers and non-emergency retailers. Unfortunately, these measures did not well stop spreading the virus. So, numerous lockdowns and stricter restrictions were imposed [8, 10].

As the COVID-19 pandemic has been challenging public health systems, and their capability to communication with the population effectively to do the best collective action [11], scholars and experts have to realize the multidimensional characteristic of risk communication (RC) and risk perception (RP) to promote community engagement in recommended behaviors and adherence to non-pharmaceutical measures as one of the most important policies and strategies to control the infection [12]. Unfortunately, no effort has been made in this field in Iran so far. However, in the previous large outbreaks, some theoretical models have been developed to explain how people perceive risks, process risk information, and adopt measures to minimize or prevent risks [13].

Since Iran like many countries around the world are experiencing the epidemic for the first time, in some case after a long time, and it is unknown that how long will the COVID-19 outbreak be with man, hence, the first hope of any government is the community engagement to promote and institutionalize non-pharmaceutical measures in order to decrease the negative impacts of epidemics on the both health and economy of societies. It is quite clear that when individuals accept such measures, they would more keen to follow and support the social regulations and uphold the requirements [6]. Many people around the world do not follow recommended behaviors such as physical distancing and this is exactly the reason why makes it difficult to control the spread of COVID-19 and shows the importance of risk communication.

Accordingly, the present research was designed to contribute to the prior research with the use of a unified procedure to explore the way through which RP mediates the relationship between RC and protective/preventive behaviors, and how RC and RP individually affect protective/preventive behaviors during the COVID-19 outbreak. To build the research structure, Health Belief Model (HBM) was employed as an overarching theoretical framework. This paper contributes to the literature via some ways. First, many of the previous researches in this field were done in post epidemics era to investigate the impact of RC on individuals’ perceptions of a risk following a disease outbreak [14, 15], while the present study was carried out in a real-time context of COVID-19 outbreak. Second, the scope of current study be broadened beyond the current focus on the one-way relationship between RC and RP in the context of emergency and outbreak situations [16, 17], and the disregarded two-way relationship be further explored [18].

### Theoretical background

#### Health belief model (HBM)

There are some theories such as the Health Belief Model (HBM), Theory of Planned Behavior (TPB), as well as Protective Motivation Theory (PMT) providing explanatory models of individuals’ reactions to threats to their health. From the beginning of the 1950s, the HBM has had extensive applications as a conceptual framework in health behavior studies, to provide explanations on the changes and continuance of health-associated behaviors and also guide the health behavior interventions [19]. During the last two decades, the HBM has experienced more expansion in comparison with other frameworks, and it consists of evaluations on the perceptions of how susceptible individuals are to diseases and how severe a disease may be. It also considers the perceived advantages as well as costs of preventive health measures along with the cues to practice [20]. Specifically, this study is grounded on this theory to explain the relationship of risk perception and protective/preventive behaviors. Given the research questions, this theory would help us to develop conceptual model.

#### Risk communication model

However, the general model of risk communication has evolved with increasing number of studies on risk communication during several years. Based on such a model, individuals perform the following actions during the risk communication process: (1) receive a warning message; (2) figure out the related content; (3) accept or believe the importance of the message included in it; (4) establish the truth of their interpretations with other people; and (5) take actions or measures regarding the message to save their lives and properties [21].

The level and type of risk communication is subject to the complexity of such a risk and the level of potential risk as well as risk perception [22]. Lundgren and McMakin (2018) has characterized three forms of risk communication as follows:

“Care communication is risk communication about health and safety risks, risks for which the danger and the way to manage it have already been well-determined through scientific research that is accepted by most of the audience.”

“Consensus communication is risk communication to inform and encourage groups to work together to reach a decision about how the risk will be managed prevented or mitigated.”

“Crisis communication is risk communication in the face of extreme, sudden danger such as the outbreak of a deadly disease.” [22].

In the case of experiencing a serious pandemic, the change of messaging takes place from support for precautions to communicating the crisis [23]. In such situation, helping organizations and agencies fulfill their mission, maintain public trust, manage limited resources, and limit harm and disruption is critical [6].

### Development of research hypotheses

A discussion on the relevant previous studies resulting in the formation of the hypotheses in this paper has been provided in this section:

#### Risk communication and risk perceptions

Many studies have focused on the associations of RC and RP [12, 17]; however, there are reports on RC influence over RP in recent research [13, 24, 25] . It is recommended that RP is a prerequisite for protective behaviors [12, 26–28]. A review of the literature reveals that there is a decrease in articles published on a one-way flow of RC as well as an increase in those concerned with two-way communications [29]; hence, RC seems to have dynamism as well as interactivity encompassing negotiations among various groups of key players and audiences [16].

If public understanding of risk is overestimated, then individuals may be thrust into situations, for the management of which they are ill-prepared. If their understanding is underestimated, then they may be disenfranchised from decisions, which could and should be made. Such misperceptions of RP may cost an arm and a leg in a long term and be expanded based on individual decisions [30]. Risk information affects individuals in complex and unpredictable ways, and the theoretical RC models are prolific across many disciplines [16]. For example, RP among the public highly depends on the way of framing the messages, the communicator of the messages, and the manner of their communication [31]. On the other hand, cross-cultural differences may pose systematic differences in RP [32], and it is RP, not a real risk, which determines the manner according to which people react to hazardous situations [31]. Consequently, at the beginning stage of a disease outbreak, at an individual level, people tend to rely on news media as a source of RC in order to assess risk; thus, the media affect how they construct their initial perception of the disease [33].

In this regard, this occurs in the presence of trust, as trust in institutions such as the government or media organizations helps reducing the complexity of and uncertainty about a particular issue [33, 34].

According to the literature, RP poses significant challenges to RC efforts [16]. Understanding how individuals perceive risks is an important factor contributing to successful RC [25]. Obviously, faulty RP may impede communications as individuals might experience misunderstandings or misinterpretations of empirical or probabilistic information [31]. On the other hand, high RP and extreme anxiety in the community may have effects on RC [16]. Accordingly, the scope of the research should be broadened beyond the current focus on the one-way relationship between RC and RP in the context of emergency and outbreak situations, and the disregarded two-way relationship should be further explored [16, 17]. A more comprehensive approach was adopted in the current paper in order to detect the relationship between RC and RP so that the following hypotheses were proposed:

**H1.** RC has significant and positive correlations with RP.

**H2.** RP has significant and positive correlations with RC.

#### Risk communication and protective/preventive behaviors

Individuals need information to make informed decisions and behave in ways that will best help them avoid risks and uncertainty [34, 35]. Nowadays, many RC activities are to change behavior or attitudes [27, 36, 37]. At the same time, RC can take place in a disengaged one-way manner as well as in a more engaged two-way manner [25].

Based on Sandman (2003) category, the risk communication in a serious pandemic is crisis communication. It is possible to regard risk communication as a means of increasing awareness, improving the knowledge or changing the behaviors as well as attitudes of engaged stakeholders, including those who have has exposure, specialists and managers, those making decisions, the public population, and media, even though it has different objectives. When a crisis is experienced, communication considerably contributes to minimization of the damages and saving lives since it affects the measures taken by all individuals involved. Accordingly, it is hypothesized that:

**H3.** RC has significant and positive correlation with protective/preventive behaviors.

#### Risk communication, risk perceptions, and protective/preventive behaviors

According to the theory of the HBM, the risk perception was suggested to be positively associated with intention to preventive/protective behavior [38]. In the field of health communication, high levels of RP make individuals be more engaged in health-protective behavioral intentions to avoid risk [13, 39]. RC semantically may have real behavioral consequences [40]. The risk communication over time leads to increase risk perception. In this way, provision of risk information can be significantly beneficial in changing risk perceptions with subsequent alteration of the impacts of risk perceptions on risk behaviors [41].

Previous studies confirm implications of risk communication practice to change of behavior, and the relationship between RP and following recommended behavior is also confirmed. For instance, Schmälzle (2017) claimed that RC may be expanded to increase RP to promote behavior [12]; thus, beyond the direct relationship between RC and RP, this study was also to examine the significance of the mediatory function of RP in the associations of the aforementioned variables and the dependent variable. Consequently, based on hypotheses 4 & 5, RP shows direct relationships with the protective/preventive behaviors, and can also mediate the association of RC and protective/preventive behaviors.

Accordingly, it is hypothesized that:

**H4.** RP has significant and positive correlations with protective/preventive behaviors.

**H5.** RP mediates the relationship between RC and protective/preventive behaviors.

## Methods

In this section, the research model is first described, and then there is a discussion about the measures adopted in the survey instruments and their validations. Finally, the sampling methods are explained along with the statistical approaches of the study.

### Research model

**Fig. 1:**
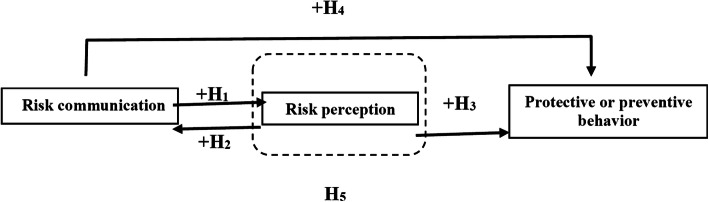
Research model and summary of the research hypotheses

Given the recent public health emergency, that is COVID-19, the community’s engagement for health protective and preventive behaviors is critical to disconnect the transmission chain. Considering the abovementioned arguments on the relationships among RC, RP, and protective/preventive behaviors, the conceptual model applied in the present paper for the examination of research hypotheses has been represented in Fig. 1.

### Instruments and measures

A cross-sectional survey was employed to collect data, obtain higher external validity, and make the findings more generalizable. RC, RP, and protective/preventive behaviors were also measured using the Likert’s scale. According to the relevant literature, four components of RC, namely news media exposure (2 items), information gathering ability (3 items), trust in the government (3 items), and trust in news media (3 items) were included in the survey. To measure RP and intention to protective/preventive behaviors, relevant items were developed using both the literature and experts’ opinions. The final questionnaire encompassed 22 items (11 RC factors, 4 RP factors, and 7 behavioral components). Moreover, some questions were aimed at gathering the respondents’ demographics while one more question addressed the main information gathering channels. In addition, one more question addressed the main information gathering channels. A blank copy of the questionnaire provides as the supplementary file 1.

### Risk communication

The RC in this study includes four components. (1) News media exposure: the study measured two groups of news media exposure, namely traditional mass media and Internet, which were evaluated with the use of a 4-point scale at a range of 1 (very often) to 4 (never) through identifying the frequency of the respondents’ exposure to news and information on COVID-19 in the mass media and Internet over the previous month (reversely coded). These items were adapted and modified based on the literature as well [15, 33, 34, 42]. (2) Information-gathering ability: three times whose scoring had been performed through a 5-point Likert scale which ranged from 1 (complete agreement) to 5 (complete disagreement) were used to measure information gathering ability. Accordingly, the participants were supposed to indicate their levels of agreement with these statements: (1) Receiving information about COVID-19 is hard for me (reversely coded); (2) I don’t know where to find information about COVID-19 (reversely coded); and (3) Even if I had access to information, it is hard for me to understand”. These items were adapted and modified based on the literature [18, 43]. (3) Trust in the Government: trust in the government was also measured using items whose scoring had been performed according to a 5-point Likert scale which ranged from 1 (complete agreement) to 5 (complete disagreement) through requiring the respondents to show their levels of agreement with these three statements: (1) I am confident that the government protects the citizens from the COVID-19 infection (reversely coded); (2) The government spare their best efforts to minimize COVID-19 infection (reversely coded); and (3) I trust in the cooperation and coordination of relevant authorities in the country (reversely coded). These items were adapted and modified based on the literature [13, 44]. (4) Trust in news media: trust in news media was evaluated using a 5-point Likert scale at a range of 1 (complete agreement) to 5 (complete disagreement) through identifying the degree of the respondents’ agreement with the three statements as follows: (1) News media provide accurate information about COVID-19 (reversely coded); (2) News media provide sufficient information about COVID-19 (reversely coded); and (3) I trust in news stories reported by news media about COVID-19 (reversely coded). These items were adapted and modified based on the literature [15, 33, 42].

### Risk perception

Measurement of RP was carried out employing a 5-point Likert scale which ranged from 1 (complete agreement) to 5 (complete disagreement) through asking how much the respondents agreed with four risk statements directly related to COVID-19: (1) COVID-19 can be serious (reversely coded); (2) I think my family and I are at risk of COVID-19 (reversely coded); (3) Iran is likely to be affected by COVID-19 (reversely coded); and (4) I trust in the usefulness of preventive measures (reversely coded).” Similarly, these items were adapted and modified based on the literature [43, 45].

### Preventive/protective behaviors

Measurement of individuals’ intention to be engaged in protective activities was carried out with the use of a 4-point Likert scale at a range of 1 (never) to 4 (very often), according to which the respondents were asked how they exhibit protective behaviors such as physical distancing, mask-wearing, good hand hygiene practices, etc. [46] through three questions. In addition, the preventive activities were also assessed with the use of a 5-point Likert scale which ranged from 1 (definitely not) to 5 (definitely), according to which the respondents had to identify the likelihood of their engagement in determined social and economic activities in the two next month through four questions: (1) socializing with others, (2) going shopping, (3) visiting entertainment venues, and (4) going sightseeing or travel. These items were adapted and modified based on the literature [13, 43].

### Questionnaire validation

The first draft of the questionnaire was submitted to five academic experts, who had expertise in the research area. Over the meetings with the experts, the validity of the questionnaires, consisting of transparency, comprehensiveness, and the correlation of the items, were evaluated. Once some questions were modified regarding their transparency and content, the final questionnaire was provided with 3 main dimensions together with 23 questions in the following stage.

### Sampling and data collection

The current study has focused on Iranians general population as the primary study population. The data for the study were derived from a nationally representative online panel survey of Iranian adults aged 15 years and above. The data were collected on March 15–19, 2020. From this online panel, a random sample was asked to participate in the survey, and 3213 persons took part in the survey, indicating a completion rate of 65%. The demographic profile of the survey respondents is presented in Table 1.

**Table 1:** Demographic profile of the survey respondents

Title	Description	Number of respondents	Percent
Age (years)	≤30	607	18.9
	31–40	1262	39.4
	41–50	816	25.5
	51–60	399	12.4
	> 60	121	3.8
Gender	Male	1591	49.5
	Female	1620	50.5
Marital status	Single	820	25.5
	Married	2391	74.5
Number of child	0	393	16.5
	1–2	1565	65.5
	3–4	385	16.1
	> 4	46	1.9
Expenditure Ratio	It’s lower	1101	34.4
	It’s balance	2103	65.6
	It’s more	0	0
Education	< Diploma	255	7.9
	Diploma	543	16.9
	Associate degree	261	8.1
	Bachelor’s degree	1081	33.6
	Master’s degree	675	21.0
	Doctoral degree	393	12.2

### Statistical approach

Confirmatory factor analysis (CFA) was performed together with a causal path analysis using structural equation modeling (SEM). To this end, after checking the normality of data distribution, the two models were tested using SEM: measurement model as well as the structural equation model. The dependence of latent variables upon or their being influenced by the observed variables can be determined through the measurement model which takes the measurement features of reliability and validity of the observed variables into account. Meanwhile, the structural equation model permits for testing the causal associations of the latent variables, illustrates direct and indirect causal effects, and defines the justified and unjustified variance [47].

In this study, application of AMOS software version 24.0 aimed at estimating and testing the proposed model and determined the causal associations. Furthermore, the model makes it possible to test the linear associations of the latent (unobserved) constructs and manifest (observed) ones. A distinguished feature of this model is its capability of making the parameter estimations available for the associations of the un-observed variables. Finally, SEM provides a path analysis which makes parameter estimations of the direct as well as indirect associations of the observed variables possible.

## Results

This section indicates the results for the reliability of the proposed measurement model, testing of hypotheses, and the analyses of mediatory effects. The results show that, 73% of Iranian people follow COVID-19 news by national mass media and social networks.

### Assessment of the measurement model reliability and validity

Composite reliability (CR), as an acceptable approach proposed by Werts et al. [48], was calculated for examination of the instrument reliability and its internal consistency. All the CR values were > 0.7. In addition, confirmatory factor analysis (First-order) aimed at testing the reliability as well as convergent validity of the three constructs. Convergent validity can be determined with regard to the factor loadings’ significance. The constructs met this prerequisite, and the factor loadings were > 0.5. The values of average variance extracted (AVE) between the construct and measures were > 0.50, implying an acceptable convergent validity. In addition, Fornell and Larcker’s [49] procedure was employed for the assessment of discriminant validity. Consequently, AVE for every construct was higher compared to the squared correlation of the construct and each of other constructs, confirming the discriminant validity of the instrument. Table 2 exhibits the details of factor loadings, critical ratios (t-value), AVE, as well as CR.

**Table 2:** Factor analysis results

Dimensions		Ref	Number of items	Factor loading	Error variances	Critical Ratio	AVE	Composite reliability
Risk Communication	News media exposure	Modified from 15, 33,34,42	2	0.68	0.54	–	0.51	0.73
				0.56	0.70	8.403		
	Information-gathering ability	Modified from 18,43	3	0.51	0.74	–	0.58	0.79
				0.82	0.33	8.281		
				0.86	0.26	7.989		
	Trust in the government	Modified from 13,44	3	0.77	0.41	–	0.64	0.85
				0.87	0.24	19.430		
				0.88	0.23	17.174		
	Trust in news media	Modified from 15,33,42	3	0.84	0.29	–	0.68	0.88
				0.83	0.31	17.706		
				0.85	0.28	18.424		
Risk Perception	Perceived severity	Modified from 43,45	4	0.82	0.33	–	0.69	0.90
	Perceived susceptibility/person & family			0.88	0.23	17.903		
	Perceived susceptibility/country			0.84	0.29	16.519		
	Perceived benefits			0.89	0.21	17.052		
Preventive/Protective Behaviors	Mask-wearing	Modified from 13,43	7	0.81	0.34	–	0.62	0.88
	Social distancing			0.83	0.31	18.851		
	Good hand hygiene practices			0.87	0.24	17.769		
	Going sightseeing or travel			0.87	0.24	17.756		
	Socializing with others			0.71	0.50	13.657		
	Going shopping			0.67	0.55	12.667		
	Visiting entertainment venues			0.77	0.41	15.026		

**Fig. 2:**
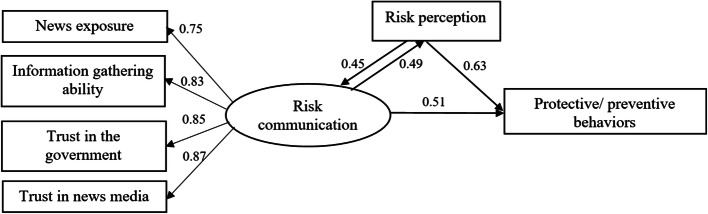
Structural equation modeling parameters

Figure 2 indicates the parameter estimations for the structural model employed in the current paper. The results of structural estimation model are represented for the validation and analysis of the research model. The results of goodness-of-fit for the statistical model describe its fitness into a series of observations, whose indices provide a summary of the discrepancies among the observed values and those predicted by the statistical model.

The indicators of absolute fit, comparative fit, as well as parsimonious fit have been presented to validate the overall fit of the model. Table 3 shows the model’s fit values in this study along with the evidence indicating that the values are within an acceptable range with regard to the guidelines and the suggested threshold.

**Table 3:** Goodness of fit measures

Type of measure	Goodness of fit measures	Definitions	Desired range	Model result	Results
Absolute fit indices	X^2^	Chi Square	> 0.05	0.072	Compliant
	GFI	Goodness of Fit Index	> 0.9	0.988	Compliant
	AGFI	Adjusted goodness of fit index	> 0.9	0.938	Compliant
	RMR	Root Mean square Residual	Near to zero	0.013	Compliant
Comparative fit indices	NNFI	Non-Normed Fit Index	> 0.9	0.989	Compliant
	NFI	Normed Fit Index	> 0.9	0.989	Compliant
	CFI	Comparative Fit Index	> 0.9	0.981	Compliant
	IFI	Incremental Fit Index	> 0.9	0.981	Compliant
Parsimonious fit indices	PRATIO	Parsimony ratio	> 0.5	0.612	Compliant
	PNFI	Parsimony Normed Fit Index	> 0.5	0.641	Compliant
	RMSEA	Root Mean Square Error of Approximation	< 0.08	0.034	Compliant
	Normed X^2^	Chi Square/ degrees of freedom	2–3	2.373	Compliant

As presented in Table 3, all the fit indicators satisfied the acceptable range recommended in the relevant studies [50, 51].

### Hypothesis testing results

The analysis results for the causal associations of the three main constructs are represented in Table 4. RC has direct as well as indirect impacts on RP (ß= 0.45 & ß =0.28, respectively), RP directly and indirectly influences RC (ß= 0.49 &ß =0.27, respectively), and RP has a direct effect on protective/preventive behaviors (ß= 0.63). Moreover, RC and protective/preventive behaviors are positively related (ß= 0.51), and RC has also an effect on protective/preventive behaviors via RP. As shown, all the relationships are statistically significant. These results indicate that risk communication promotes risk perception, and subsequently the appropriate perception of risk improves risk communication. These results support the mutual relationship between RC and RP (H_1_ and H_2_). The correlation between RP and protective/preventive behaviors supports the direct correlation of RP and protective/preventive behaviors (H_3_). Eventually, path analysis was used to examine the Overall impacts of RC on RP and protective/preventive behaviors. As presented in Table 4, the impact of RC as an exogenous variable on protective/preventive behaviors is statistically significant with regard to H_4_, and the stability index is 0.334 for RC and RP.

**Table 4:** SEM results for hypothesis testing

Hypothesis		Path coefficient	C.R. (t-value)	Result
H_1_	RC >>> RP	0.45	13.32**	Supported
H_2_	RP >>> RC	0.49	13.75*	Supported
H_3_	RP >>> Behaviors	0.63	11.47***	Supported
H_4_	RC >>> Behaviors	0.51	14.23***	Supported

*Denotes *p* < 0.05. ** Denotes *p* < 0.01. *** Denotes p < 0.001

### Mediatory effects

According to what was mentioned above, the direct and indirect effects of RC on protective/preventive behaviors were examined using two different approaches. In this respect, the results of the study indicate significant and positive contribution of RC to RP (direct effect = 0.45, *p* < 0.001). Furthermore, RP has positive effects on protective/preventive behaviors (direct effect = 0.63, *p* < 0.0001), and RC significantly contributes to protective/preventive behaviors (direct effect = 0.51, p < 0.0001). The indirect effect of RC on protective/preventive behaviors with regard to the mediating role of RP increased by 0.84. Consequently, according to Baron and Kenny [52], RP plays a mediating role in the relationship between RC and protective/preventive behaviors. This finding supports H_5_. The Table 5 shows the mediating impact of RP on the association between RC and Protective/preventive behaviors (H5).

**Table 5:** Effects of RC on RP and Protective/preventive behaviors

Hypothesis	Direct effect			Indirect effect		
	Path coefficient	t-value	P	Path coefficient	t-value	P
RC >>> RP	0.492	6.396	***			
RP >>> RC	0.448	5.436	***			
RC >>> RP >>> RC				0.276	4.115	***
RP >>> RC >>> RP				0.269	4.078	***
RP >>> Behaviors	0.633	8.647	***			
RC >>> Behaviors	0.508	7.128	***			
RC >>> RP >>> Behaviors				0.328	5.221	***

***Denotes p < 0.0001

## Discussion

Risk communication plays a critical role in an effective communication and supports public needs under stressful situations [25]. This study contributes to our knowledge of how the risk communication affects protective and preventive behaviors during the global epidemic of the COVID-19. The findings of this study provide a comprehensive understanding of how people shape their perceptions of risk with regard to risk communication that leads to behavioral intentions in public health risk situations. The findings show that risk communication both determines and influences a thorough understanding of risk perceptions, and this is an issue ignored in previous studies. Besides, the mediatory contribution of risk perception in the association of risk communication and protecting/preventing behaviors is confirmed in the present study.

In all major global public health events in this new millennium, including outspread of severe acute respiratory syndrome (SARS), Middle East respiratory syndrome (MERS), influenza A(H1N1), as well as Ebola, RC and community engagement were integral to the success because RC determines which hazards people care about and how they deal with them [11].

The positive effect of risk perception on protective behavior has been approved in previous studies on COVID-19 [43, 53–55] and other infectious diseases [12, 24, 56]. For example, the reactions against the A/H1N1 virus outspread in 2009 show the considerable importance of individuals’ perceptions of risk in achieving positive results from public health intervention programs [24]. As Weinstein mentioned, exact perceptions of risk can allow people for making proper decisions regarding the actions which help prevent illnesses or injuries [57].

Risk communication need to be concentrated mainly on communication of risk-mitigation alternatives considered as useful by the target audience [26], identifying group-specific demands would be beneficial to render proper information to meet each population group’s needs [58]. Thus, the mutual relationship between risk communication and the status of risk perceived by community members is of great importance. This was exactly addressed in this study, i.e. the reciprocal relationship between RC and RP. In this regard, intensive media coverage about COVID-19 would contribute to amplifying COVID-19 risks and increasing the public’s risk perception to enhance the capacity of those people in rapidly responding to the COVID-19 epidemic [59]. From this perspective, the greater exposure to news about the COVID-19 outbreak is expected to increase the public RP.

Previous research provides evidence to assume that social media help individuals during risks and crises [60]. For instance, Dijl et al. showed that social media increased information sufficiency and decreased insecurity of what was happening. Accordingly, these positive effects on “knowing what is going on” could cause more awareness about what might be an appropriate measure to be adopted [61]. Accordingly, it seems inherently plausible that people need not only to be aware of an the existing health risks but also to feel themselves at risk in order to adopt protective measures [24].

Information-gathering capability would be particularly important during the COVID-19 outbreak because the disease is unfamiliar, generating excessive public concerns and uncertainty. An individual’s information-gathering ability can promote his/her confidence in processing risk-related information more systematically [14, 15, 35, 44]. Previous studies on the risk communication have regarded self-efficacy primarily as the individuals’ sense of their own abilities to address the media and the information related to the risks [18]. Information searching is essential to achieve the determined consequences, particularly when active non-routine information is collected through various information channels [18].

In the case of widespread societal public health issues such as the COVID-19 outbreak, about which individuals have no firsthand experience, they tend to trust in social media and institutions [62]. This trust might exert a significant influence on the public RP [14]. In other words, individuals with higher levels of confidence in government face higher possibilities to perceive that the government has sufficient ability and knowledge to deal with the crisis. Thus, the higher levels of trust in the government can significantly lead to the implementation of the proposed instructions and requirements. In this regard, You and Ju (2015) mention that media are the initial information resources for the public in a health issue [63]. Similarly, trust in news media can also influence the public RP. When people trust in the risk information published by the media, their uncertainty toward the risk decreases [13]. Trust enables individuals to judge the risk in the absence of complete knowledge or understanding [62]. Low efficacious messages would not meet individuals’ needs in the crisis and makes them turn elsewhere [28].

According to the protective motivation theory [64], RP can motivate behavior since individuals activate protection motivation to prevent negative outcomes when they perceive risks. In a risky environment, such individuals are more likely to take actions to reduce the threat or avoid the danger. For example, when an infectious disease outbreak occurs, they exhibit physical distancing behavior. That is, they decrease their social contacts to protect their health and are reluctant to engage in social or economic activities such as socializing with friends, sightseeing, and shopping, as observed in the South Korean during MERS outbreak [13]. In the previous studies on disastrous events and emergencies, the determining factors of RP and behavior are message features and their processing by the receivers [31]. As it is found, if the risk communication fails to convey the severity of a risk correctly, it will be less likely to influence the appropriate behaviors. This means that we need to use the fresh knowledge of risk communication research in order to develop an appropriate risk perception among individuals in the time of emergency.

On the other hand, if we consider risk perception as an emotional response, according to Slovic (2006), individuals show higher likelihood to carry out adaptive behaviors in the case of stronger emotions, at presence of some perceived social pressure, and with higher quality of the warning messages. This finding supports the significance of affective reactions as stimulators of behavior [65]. Although cognitive factors such as the evaluated quality of the message are clearly essential for a behavior, Gutteling et al. noted that emotions and social environment are the primary predicting factors for the actual performance of the required behavior [27]. When a warning message is received, the higher the risk is perceived, the higher will be the level of emotions, and more perceived expectations from individual’s social circumstances are supposed to bring about higher avoidance probabilities. Further avoidance behaviors and higher frequency of emotions as well as perceived risk appear to be consistent with previous studies [27].

The present work had some limitations which are worth mentioning. First, although the research employed nationally representative survey data to study the impact of risk communication on the preventive and protective actions in COVID-19 outbreak, the findings of this study cannot be generalized to the whole country. Furthermore, only correlations but not the causal relationships were explored due to the cross-sectional design of this study.

## Conclusion

The findings of the study have primarily indicated that risk communication cannot be regarded as a passive and one-way procedure any more, and that it has a direct relationship with risk perception. Therefore, there needs to be a consensus on an active reciprocal approach, in which appropriate behaviors are strengthened and developed, and people perceive individual responsibility to take preventive and protective actions. Risk communication focusing on the promotion of self-protectiveness can be influential if there are correct risk perception conditions. An effective risk communication campaign has to focus on the effectiveness of risk-mitigation actions and ensure that this communication is perceived actively. Thus, risk communication should be carefully established and planned across the lines of behavioral actions, which are considered efficient and reliable by a considerable number of individuals in the target audience.

## Supplementary Information

**Additional file 1 **The questionnaire: A blank copy of the questionnaire**.**

## Data Availability

The datasets used and/or analyzed during the current study available from the corresponding author on reasonable request.

## Abbreviations

**AVE**: Average Variance Extracted
**CFA**: Confirmatory Factor Analysis
**COVID-19**: The new coronavirus
**CR**: Composite reliability
**HBM**: Health Belief Model
**H1N1**: Influenza A
**MERS**: Middle East Respiratory Syndrome
**PMT**: Protective Motivation Theory
**PPB**: Protective and Preventive Behaviors
**RC**: Risk Communication
**RP**: Risk Perception
**SARS**: Severe Acute Respiratory Syndrome
**SEM**: Structural Equation Modeling
**TPB**: Theory of Planned Behavior
