# Supplementary material for: The effect of risk communication on preventive and protective Behaviours during the COVID-19 outbreak: mediating role of risk perception
Source: BMC Public Health. 2021 Jan 6;21:54. doi: 10.1186/s12889-020-10125-5 (PMC7787415; doi:10.1186/s12889-020-10125-5)
Supplement: Supplementary file 1 — Additional file 1 The questionnaire: A blank copy of the questionnaire. [file 12889_2020_10125_MOESM1_ESM.docx]

**The esteemed participant,**

My name is …. The present questionnaire aims to contribute to the existing knowledge on how risk communication (RC) and risk perception (RP) affect protective and preventive behaviors (PPB) during the COVID-19 outbreak. It takes approximately 15 minutes to complete this questionnaire. Your participation in this study is entirely voluntary. The information and data are strictly confidential. We will publish only aggregated, anonymous results.

Thank you for your support!

**Answer Guide:**

Please, choose the most suitable option in your opinion.

Table Q.1: Demographic profile.

| **Age (years):** |
| --- |
| **Gender:** |
| **Marital status:** |
| **Number of child:** |
| **Education:** |
| **Expenditure Ratio:**  **⃝** Expenditure> Income  ⃝ Expenditure= Income  ⃝ Expenditure< Income |
| **Tel/ Phone (Optional):** |
| **How do you mostly follow Corona/COVID-19 news?**  **⃝** National TV  ⃝ Foreign media/ satellite  ⃝ Social networks (WhatsApp, Telegram, Facebook, etc.)  ⃝ Internet  ⃝ Telephone/ SMS  ⃝ Newspapers and publications |

Table Q.2: The questionnaire.

| 1. **Risk communication** | | | | | |
| --- | --- | --- | --- | --- | --- |
| **A: News Media Exposure** | | | | | |
|  | Never | Seldom | Sometimes | Very often | |
| How many times a day have you followed the news and information on COVID-19 in the mass media on average during the last month? |  |  |  |  | |
| How many times a day have you followed the news and information on COVID-19 in the Internet over the previous month? |  |  |  |  | |
| **B: Information-Gathering Ability** | | | | | |
|  | Completely disagree | Disagree | Undecided | Agree | Completely agree |
| Receiving information about COVID-19 is hard for me. |  |  |  |  |  |
| I don’t know where to find information about COVID-19. |  |  |  |  |  |
| Even if I had access to information, it is hard for me to understand. |  |  |  |  |  |
| **C: Trust in the Government** | | | | | |
|  | Completely disagree | Disagree | Undecided | Agree | Completely agree |
| I am confident that the government protects the citizens from the COVID-19 infection. |  |  |  |  |  |
| The government spare their best efforts to minimize COVID-19 infection. |  |  |  |  |  |
| I trust in the cooperation and coordination of relevant authorities in the country. |  |  |  |  |  |
| **D: Trust in News Media** | | | | | |
|  | Completely disagree | Disagree | Undecided | Agree | Completely agree |
| News media provide accurate information about COVID-19. |  |  |  |  |  |
| News media provide sufficient information about COVID-19. |  |  |  |  |  |
| I trust in news stories reported by news media about COVID-19. |  |  |  |  |  |
| 1. **Risk perception** | | | | | |
|  | Completely disagree | Disagree | Undecided | Agree | Completely agree |
| COVID-19 can be serious. |  |  |  |  |  |
| I think my family and I are at risk of COVID-19. |  |  |  |  |  |
| Iran is likely to be affected by COVID-19. |  |  |  |  |  |
| I trust in the usefulness of preventive measures. |  |  |  |  |  |
| 1. **Protective/preventive behaviors** | | | | | |
| **A: Protective behaviors** | | | | | |
|  | Never | Seldom | Sometimes | Very often | |
| How much do you use protective equipment such as masks, gloves, etc.? |  |  |  |  | |
| How much do you observe your 1-2 meter distance with others? |  |  |  |  | |
| Do you wash your hands regularly during and after being outside? |  |  |  |  | |
| **B: Preventive behaviors** | | | | | |
|  | Definitely | Very Probably | Possibly | Probably not | Definitely not |
| Will you socializing with others in the two next month? |  |  |  |  |  |
| Are you going shopping (other than buying the basic necessities of life) in the two next month? |  |  |  |  |  |
| Are you visiting entertainment venues in the two next month? |  |  |  |  |  |
| Are you going sightseeing or travel in the two next month? |  |  |  |  |  |

Thank you very much for your participation.
